# Commentary: Preemptive left ventricular unloading in high-risk cardiac operations—uncertain risk/benefit relationships, but promising!

**DOI:** 10.1016/j.xjon.2021.10.043

**Published:** 2021-10-27

**Authors:** Victor A. Ferraris

**Affiliations:** Lexington VA Medical Center and Department of Surgery, University of Kentucky, Lexington, Ky

**Figure:**
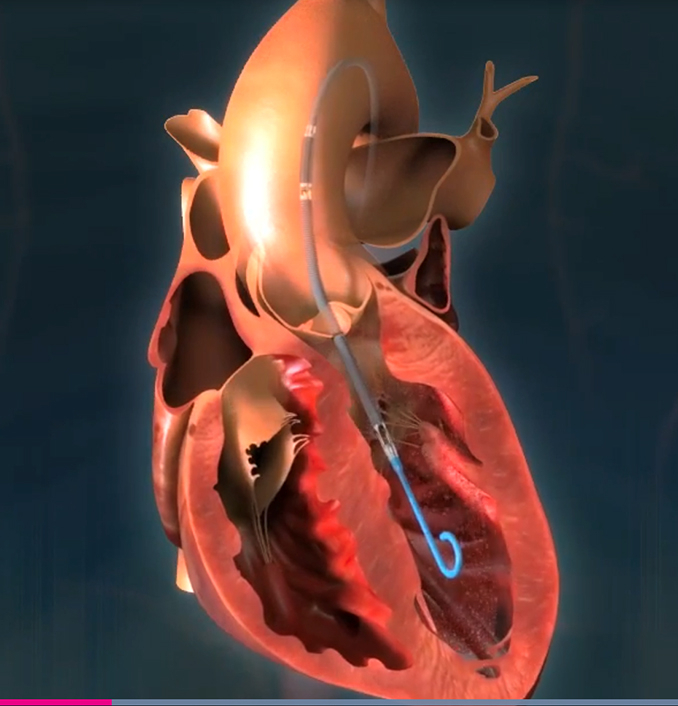
Preemptive left ventricular unloading in high-risk cardiac operations may prove beneficial.

**Central Message**

Preemptive left ventricular unloading with an Impella device may have benefit for high-risk patients with left ventricular impairment during high-risk cardiac operations.

See Article page 10.

Goldstein and Soltesz^1^ present a preliminary report on the use of the advanced Impella support device (Impella version 5.5; Abiomed, Danvers, Mass) for preemptive ventricular support in high-risk patients undergoing operation in the setting of significant ventricular compromise. When preexisting stunned, hibernating, and/or acutely ischemic myocardium is subjected to the pathophysiology of the cardiopulmonary bypass circuit, to the ischemia mandated by crossclamping, and to the possibility of suboptimal cardioprotection, the result is often a dangerous combination of vasoplegia and postoperative low cardiac output. Treatments for this set of circumstances have traditionally involved inotropes and intra-aortic balloon pump support. More invasive options, ranging from extracorporeal membrane oxygenation to more elaborate ventricular support interventions, often follow if these support options are ineffective. Consensus suggests that extracorporeal membrane oxygenation support is not a perfect option in the setting of extreme postbypass ventricular dysfunction. Similarly, more extreme forms of ventricular support are high-risk undertakings for patients who have failed other support methods. Other rather extreme measures like left ventricular assist devices and transplantation are final steps in this decision algorithm and these are even less likely to provide a satisfactory outcome in the urgent setting of low cardiac output and severe left ventricular dysfunction after operation. In response to postcardiotomy shock, Goldstein and Soltesz^1^ present an intriguing option: The paradigm of preventive support with preemptive ventricular unloading.

The authors start by suggesting that reliable information and prediction models for postcardiotomy shock development do not exist. They suggest—emphasis on suggest—that preemptive ventricular unloading may limit ventricular dysfunction after cardiopulmonary bypass and provide support to allow reparative operative interventions to provide improved recovery and enhanced postoperative survival. Their choice for providing this preemptive ventricular support is implantation of an Impella device. Their hypothesis is that the preemptive ventricular unloading with this device during high-risk cardiac operations may limit intraoperative myocardial damage and allow improved surgical outcomes in high-risk patients.

To be fair, the current Impella device (version 5.5) has not been used for, and is not approved for, this indication. The prophylactic use of the Impella device to limit left ventricular dysfunction during and following high-risk cardiac operations would be a new use of this device. The authors have skirted this issue a bit, perhaps for understandable reasons. They acknowledge that the Impella device has not been used in a prophylactic manner. Further, the conduct of a randomized trial to test the effectiveness of the Impella device as a prophylactic intervention would be a statistical design nightmare clouded with ethical issues and uncertain indications for use. Goldstein and Soltesz^1^ are aware of these issues and acknowledge that the published experience with the Impella device is limited to 2 reports that studied a cohort of slightly more than 200 patients, nearly all of whom did not have device insertion for prophylactic purposes.

The use of the Impella device for prophylaxis in certain high-risk patients is a novel, appealing idea that needs further exploration. The exact path forward is complicated, but it appears that traditional pathways for approval of a new device indication are likely to be too cumbersome and may not be necessary. More evidence may only be obtained by experiential observations and anecdotal reports. This is a new pathway in the modern era that may be the best option.
